# Association between gestational weight gain in women with gestational diabetes mellitus and adverse pregnancy outcomes: a retrospective cohort study

**DOI:** 10.1186/s12884-021-03982-4

**Published:** 2021-07-14

**Authors:** Ping Shi, Aimin Liu, Xiaoyan Yin

**Affiliations:** 1 Wujin Hospital Affiliated With Jiangsu University, Tianning District, No 2 Yongning North Road, Changzhou, Jiangsu China; 2 The Wujin Clinical College of Xuzhou Medical University, Tianning District, No 2 Yongning North Road, Changzhou, Jiangsu China

**Keywords:** Gestational weight gain, Gestational diabetes mellitus, Institute of Medicine guidelines, Adverse pregnancy outcomes

## Abstract

**Background:**

To examine association between gestational weight gain (GWG) in women with gestational diabetes mellitus (GDM) and adverse pregnancy outcomes (APOs).

**Methods:**

This retrospective cohort study enrolled women with GDM who delivered at 2010–2020 in Changzhou, Jiangsu. Total GWG, rates of GWG in second trimester and third trimesters were stratified into three categories according to IOM guidelines: within, below, and above IOM guidelines. Univariable and multivariable logistic regression analyses were used.

**Results:**

Overall, 1606 women with GDM fulfilled inclusion criteria. Compared with within IOM guidelines and after adjusting for confounders, total GWG above IOM guidelines in pregnancy was associated with an increased odds of caesarean delivery [adjusted odds ratio (aOR) = 1.34, 95% confidence interval (CI): 1.04–1.72], hypertensive disorders of pregnancy (HDP) (aOR = 2.00, 1.28–3.12), preeclampsia (aOR = 2.06, 1.01–3.12), macrosomia (aOR = 1.55, 1.13–2.13) and large for gestational age (LGA) (aOR = 2.82, 1.94–3.23), and a decreased odds of premature rupture of membrane (PROM) (aOR = 0.46, 0.36–0.60) and preterm birth (aOR = 0.35, 0.26–0.44); total GWG below IOM guidelines in pregnancy was associated with an increased risk of preterm birth (aOR = 1.96, 1.44–2.66), small for gestational age (SGA) (aOR = 1.32, 1.11–1.50) and a decreased odds of macrosomia (aOR = 0.35, 0.23–0.53) and LGA (aOR = 0.54, 0.42–0.72). Further, in both second and third trimesters of pregnancy, rates of GWG above IOM guidelines was found to be associated with a high odds of HDP (aOR = 2.55, 1.86–3.38; aOR = 1.93, 1.08–2.98), preeclampsia (aOR = 2.28, 1.21–3.81; aOR = 2.17, 1.35–4.37), macrosomia (aOR = 1.20, 1.02–1.82; aOR = 2.02, 1.51–2.64) and LGA (aOR = 1.42, 1.24–1.97; aOR = 1.79, 1.51–2.54). Rates of GWG above IOM guidelines in third trimester of pregnancy also increased odds of caesarean delivery (aOR = 1.48, 1.16–2.34) when compared with within IOM guidelines. While rates of GWG below IOM guidelines in both second and third trimesters of pregnancy was associated with a decreased odds of macrosomia (aOR = 0.66, 95% CI: 0.52–0.78; aOR = 0.52, 0.39–0.63) and LGA(aOR = 0.71, 0.51–0.82; aOR = 0.67, 0.55–0.79). In addition, rate of GWG below IOM guidelines in third trimester of pregnancy was associated with an increased odds of preterm birth (aOR = 1.52, 1.12–2.05) and SGA (aOR = 1.21, 1.10–1.69).

**Conclusion:**

GWG, outside IOM guidelines has increased risks of APOs among women with GDM, implying that careful surveillance for GWG during different stages of pregnancy is warranted.

## Background

Gestational diabetes mellitus (GDM) is defined as hyperglycaemia first detected during pregnancy and not reaching non-pregnant diabetes levels; it is one of the major risk factors of adverse pregnancy outcomes (APOs) [1, 2]. Previous studies have shown that the diagnosis of GDM was highly associated with a risk of hypoglycemia, hyperbilirubinemia, preeclampsia, and cesarean section. It was also associated with a risk of fetal macrosomia, preterm birth, and large for gestational age (LGA) infants, in addition, women with GDM were shown to be at a risk of long-term obesity and diabetes [3, 4]. In China, the prevalence of GDM is approximately 10% of pregnancies [5]. However, recent studies have shown that the prevalence of GDM has increased with both obesity and gestational weight gain (GWG) among pregnant women on other areas of the world and has been correlated with APOs [6, 7].

GWG, an important antenatal factor, is reportedly associated with APOs [8–10]. According to the 2009 Institute of Medicine (IOM) guidelines, excessive GWG increases the risk of cesarean delivery, hypertensive disorders of pregnancy (HDP), GDM, and LGA infants. On the other hand, insufficient GWG increases the risk of small for gestational age (SGA) infants and preterm births [11–14]. However, studies on the association of GWG with APOs in women with GDM are conflicting [15–18]. Yasuda et al. indicated that excessive GWG in women with GDM was significantly related to increased infant birthweight [19]. Insufficient GWG reportedly increased the incidence of preterm birth in women with GDM [20]. Moreover, some studies showed that insufficient GWG in women with GDM is associated with more favorable obstetric and neonatal outcomes than adequate or excessive GWG [16, 21]. However, whether inadequate GWG is associated with adverse outcomes in GDM has not been fully elucidated. Additionally, there is limited research on the association of APOs with GDM among the adequate range of GWG at different stages (total GWG and rates of GWG in the second trimester and third trimesters).

Therefore, we conducted a retrospective cohort study of women with GDM in China which included a wide range of perinatal outcomes to investigate the associations among GWG within, below, or above the IOM guidelines with adverse perinatal outcomes. We also assessed whether inadequate GWG is associated with adverse outcomes in GDM in the second and third trimesters of pregnancy.

## Methods

### Study design and population

The study was conducted at a hospital in Changzhou, Jiangsu, China. We analyzed the data recorded for pregnant women diagnosed with GDM who delivered between January 2010 and December 2020, all data were extracted from the institutional medical record database. All pregnant women self-reported their pre-gestational body mass index (ppBMI) and measured their height and weight at the first trimester visit to the hospital. Their weight was then recorded in the electronic medical records during every subsequent antenatal clinical visit to the hospital. According to the following World Health Organization (WHO) classifications for body mass index (BMI), women were classified into four categories by their ppBMI (see Table 1). All pregnant women had their last weight measured at delivery within 24 h of entry into the labor room. Total GWG was calculated by deducting pre-gestational weight from maternal weight at delivery; The rates of GWG were calculated by dividing the GWG in the second ( 16 weeks) or third trimester (final number of weeks) by the corresponding number of weeks. The GWG of different stages (total GWG and rates of GWG in the second trimester and third trimesters) was stratified into three categories according to IOM guidelines: within, below, and above the IOM guidelines (see Table 1) [22].

**Table 1:** IOM guidelines for total GWG and rates of GWG during Pregnancy, by ppBMI

	Total GWG (kg)	Rates of GWG in second and third Trimester (kg/week)
Underweight (< 18.5 kg/m^2^)	12.5–18	0.44–0.58
Normal weight (18.5–24.9 kg/m^2^)	11.5–16	0.35–0.50
Overweight (25.0–29.9 kg/m^2^)	7–11.5	0.23–0.33
Obese (≥ 30.0 kg/m^2^)	5–9	0.17–0.27

*IOM* Institute of Medicine, *GWG* gestational weight gain, *ppBMI* pre-pregnancy Body Mass Index

**Fig. 1:**
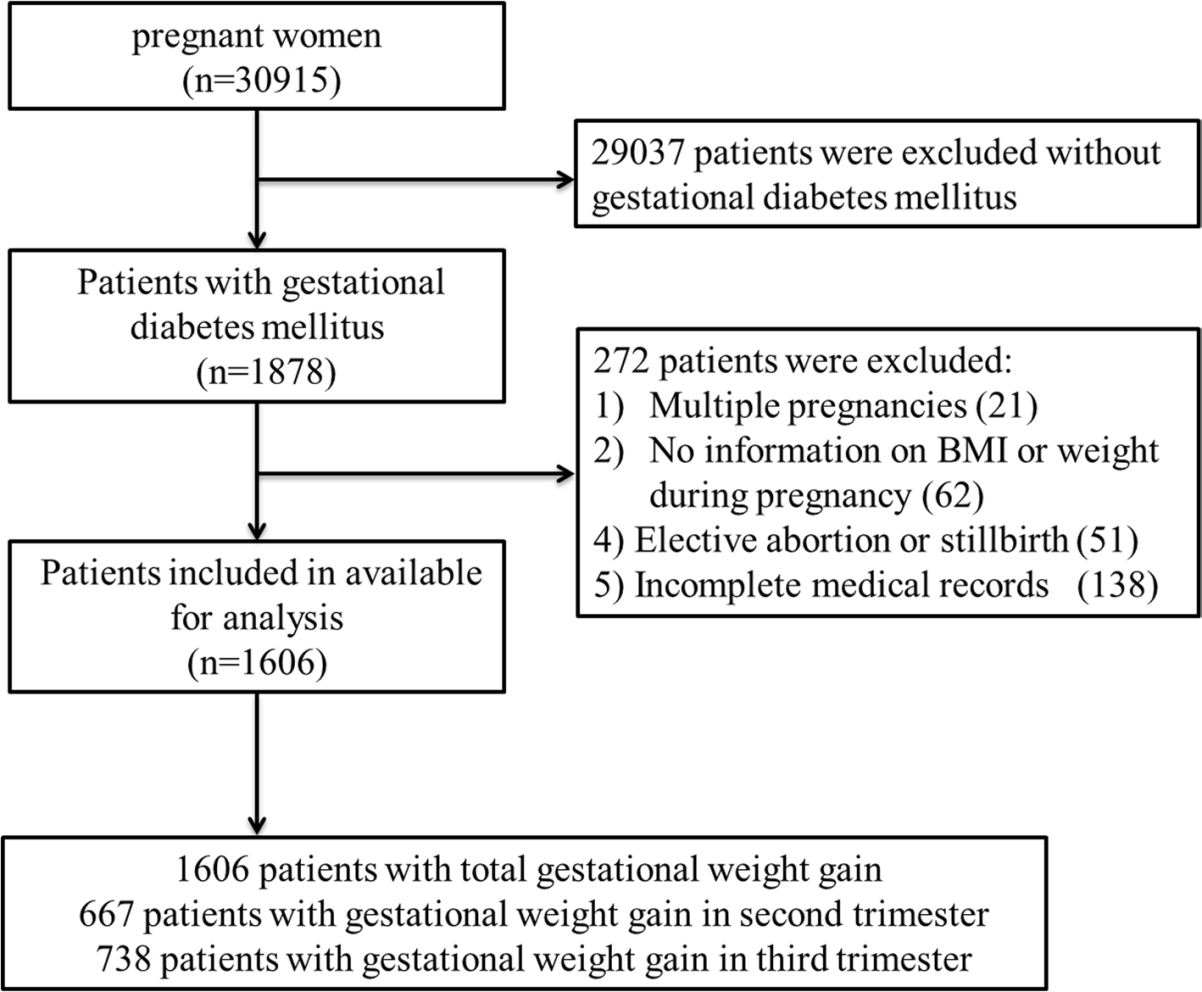
Flow diagram of Study cohort

The inclusion criteria comprised of (a) aged 18 years or older without pre-GDM, pre-gestational hypertension, heart disease, hepatic disease, or renal disease; (b) diagnosed with GDM with singleton pregnancy and live birth; and (c) complete medical records of APOs. The exclusion criteria were as follows: (a) multiple pregnancies; (b) no information on ppBMI or weight during pregnancy; and (c) elective abortion or stillbirth before 22 weeks of pregnancy. A total of 30,915 pregnant women delivered at our hospital; 1878 pregnant women with GDM were included after applying the inclusion criteria. However, 272 of them were removed from the study after applying the exclusion criteria (Fig. 1). Finally, 1606 pregnant women with GDM were included for analysis. Data of general information (included maternal age, pre-pregnancy BMI, maternal education, caesarean history, parity, IVF, mode of delivery, gestational age), gestational weight, blood glucose, and APOs were obtained from the institutional medical record system. The APOs consisted of adverse maternal pregnancy outcomes and adverse neonatal outcomes. We examined the following adverse maternal pregnancy outcomes: cesarean delivery, HDP, preeclampsia, premature rupture of membranes (PROM), postpartum hemorrhage, and fetal distress. Adverse neonatal outcomes examined were preterm birth (before 37 weeks of pregnancy), macrosomia with birth weight ≥ 4000 g, SGA (birth weight below the10^th^ percentile per gestational age and gender), and LGA (birth weight above the 90^th^ percentile per gestational age and gender).

### Diagnosis of GDM

GDM was diagnosed when any blood glucose value was greater than fasting blood glucose at 5.1 mmol/L or blood glucose after 1 h at 10.0 mmol/L or after 2 h at 8.5 mmol/L based on a 75-g oral glucose tolerance test (75 g OGTT). This was based on the criteria of the International Association of Diabetes and Pregnancy Study Groups (IADPSG) [23].

### Statistical analysis

Statistical analysis of all data was completed using SPSS version 23.0 (SPSS, Chicago, IL, USA). Mean ± standard deviation (SD) was used to describe continuous variables, and categorical data were expressed as proportions (n [%]). The ANOVA test were used to analyze the differences in continuous variables, and Pearson’s χ2 test or Fischer exact test were used to analyze categorical data. Adjusted odds ratios (aORs) with 95% confidence intervals (CIs) were calculated to express the odds ratios of the GWG above or below the IOM guidelines on APOs by multivariable logistic regression after adjusting for potential confounding variables. A *p* value < 0.05 (two sided) indicated statistical significance.

## Results

### Demographic characteristics of the cohort

Overall, 1606 women were enrolled according to the inclusion criteria (Fig. 1). The GWG of 560 women (34.9%) was within the IOM guidelines, 545 (33.9%) had GWG below IOM guidelines, and 501 (31.2%) had GWG above the IOM guidelines. The demographic and clinical data of these three groups are summarized in Table 2. The pregnant women in the GWG below IOM guidelines group were significantly older than those in the GWG within and above IOM guidelines group (mean age ± SD: 33.21 ± 4.36 vs 32.75 ± 4.83 vs 31.86 ± 4.58 years, *P* < 0.01). The ppBMI in the above IOM guidelines group (23.68 ± 3.41 kg/m^2^) was significantly higher than that in the within (22.72 ± 3.11 kg/m^2^) and below (22.48 ± 2.62 kg/m^2^) IOM guidelines groups. The proportion of overweight and obese women (25.2%) was highest in the above IOM guidelines group, and women in the below IOM guidelines group had the highest proportion (81.7%) of normal ppBMI. In addition, there were significant differences in the mode of delivery, maternal education, fasting plasma glucose (FBG), and 2 h GLU among the three groups. However, there were no significant differences in cesarean history, parity, or in-vitro fertilization in these three groups (*P* > 0.05) (see Table 2).

**Table 2:** Characteristics of women with gestational diabetes mellitus stratified by GWG according to IOM guidelines

Characteristic	Within (*n* = 560) (34.9%)	Below (*n* = 545) (33.9%)	Above (*n* = 501) (31.2%)	*P* value
Maternal age	32.75 ± 4.83	33.21 ± 4.36	31.86 ± 4.58	< 0.001
Pre-pregnancy BMI (kg/m^2^)	22.72 ± 3.11	22.48 ± 2.62	23.68 ± 3.41	< 0.001
BMI category [n (%)]				< 0.001
Underweight (< 18.5)	68(12.1)	60(11.0)	37(7.4)	
Normal weight (18.5–24.9)	414(73.9)	445(81.7)	338(67.5)	
Overweight (25–29.9)	71(12.7)	37(6.8)	108(21.6)	
Obese (≥ 30)	7(1.3)	3(0.6)	18(3.6)	
Maternal education [n (%)]^a^				0.002
Low	2(0.4)	2(0.4)	6(1.2)	
Medium	100(17.9)	76(13.9)	112(22.4)	
High	458(81.8)	467(85.7)	383(76.4)	
Caesarean history [n (%)]	105(18.8)	90(16.5)	96(19.2)	0.481
Parity [n (%)]				0.869
Primiparous	330(58.9)	327(60.0)	303(60.5)	
Multiparous	230(41.1)	218(40.0)	198(39.5)	
IVF [n (%)]	51(9.1)	55(10.1)	54(10.8)	0.657
Mode of delivery [n (%)]				< 0.001
Vaginal	325(58.0)	346(63.5)	254(50.7)	
Cesarean	235(42.0)	199(36.5)	247(49.3)	
Gestational age (weeks, mean ± SD)	38.16 ± 2.00	37.49 ± 2.46	38.61 ± 1.68	< 0.001
FPG on OGTT (mmol/L)	5.03 ± 0.95	5.53 ± 0.78	5.62 ± 0.89	< 0.001
2-h blood glucose on OGTT (mmol/L)	8.83 ± 1.71	8.72 ± 1.65	8.49 ± 1.58	< 0.001

*IOM* Institute of Medicine, *GWG* gestational weight gain, *BMI* Body Mass Index, *IVF* in-vitro fertilization, *SD* standard deviation, *FPG* fasting blood glucose, *OGTT* oral glucose tolerance test; ^a^ Low, primary school or less; Medium, middle and high school graduate; High, College or above

### Association of total GWG with APOs among women with GDM

We analyzed the APOs according to total GWG among women with GDM. In comparison to pregnant women with GWG within the IOM guidelines, women with total GWG above the IOM guidelines had an increased odds of cesarean delivery (aOR = 1.34; 95% CI 1.04–1.72), HDP (aOR = 2.00; 95% CI 1.28–3.12), preeclampsia (aOR = 2.06; 95% CI 1.01–3.12), macrosomia (aOR = 1.55; 95% CI 1.13–2.13), and LGA (aOR = 2.82; 95% CI 1.94–3.23). Conversely, they had a decreased odds of PROM (aOR = 0.46; 95% CI 0.36–0.60) and preterm birth (aOR = 0.35; 95% CI 0.26–0.44).

Women with total GWG below the IOM guidelines had an increased odds of preterm birth (aOR = 1.96; 95% CI 1.44–2.66) and SGA (aOR = 1.32; 95% CI 1.11–1.50). However, they had a decreased odds of macrosomia (aOR = 0.35; 95% CI 0.23–0.53) and LGA (aOR = 0.54; 95% CI 0.42–0.72). There were no statistically significant differences in the odds ratios of postpartum hemorrhage, fetal distress, and placenta previa among all three groups (Table 3).

**Table 3:** Association of adverse pregnancy outcome among gestational diabetes mellitus with IOM guideline on total GWG

	Within (*n* = 560)	Below (*n* = 545)			Above (*n* = 501)		
	n(%) (Reference)	n (%)	crude OR (95% CI)	Adjusted OR (95% CI)	n (%)	Crude OR (95% CI)	Adjusted OR (95% CI)
Caesarean delivery	235(42.0)	199(36.5)	0.80(0.62–1.01)	0.79(0.61–1.01)	247(49.3)	1.35(1.06–1.71)^a^	1.34(1.04–1.72)^b^
HDP	36(6.4)	25(4.6)	0.70(0.41–1.18)	0.63(0.406–1.11)	65(13.0)	2.17(1.15–3.32)^b^	2.00(1.28–3.12)^b^
preeclampsia	15(2.7)	11(2.0)	0.80(0.35–1.85)	0.83(0.35–1.97)	33(6.6)	2.30(1.17–4.53)^b^	2.06(1.01–4.21)^b^
PROM	263(47.0)	278(51.0)	1.18(0.93–1.49)	1.17(0.92–1.48)	147(29.3)	0.47(0.36–0.61)^a^	0.46(0.36–0.60)^b^
Postpartum hemorrhage	50(8.9)	58(10.6)	1.22(0.82–1.81)	1.24(0.83–1.85)	43(8.6)	0.96(0.63–1.47)	0.90(0.58–1.40)
Fetal distress	164(29.3)	152(27.9)	0.93(0.72–1.21)	0.93(0.72–1.22)	138(27.5)	0.92(0.71–1.20)	0.94(0.71–1.23)
Preterm birth	89(15.9)	138(25.3)	2.39(1.63–2.42)^a^	1.96(1.44–2.38)^a^	34(6.8)	0.39(0.25–0.58)^a^	0.35(0.23–0.44)^a^
Macrosomia	90(16.1)	33(6.1)	0.34(0.22–0.55)^a^	0.35(0.23–0.53)^a^	123(24.6)	1.70(1.25–2.30)^a^	1.55(1.13–2.13)^b^
SGA	41(7.3)	59(10.8)	1.43(1.23–1.69)^a^	1.32(1.11–1.50)^b^	21(4.2)	0.66(0.51–0.87)^b^	0.89(0.69–1.09)
LGA	126(22.5)	73(13.4)	0.47(0.35–0.68)^a^	0.54(0.42–0.72)^a^	183(36.5)	3.04(2.12–4.25)^a^	2.82(1.94–3.23)^a^

Multivariate analyses were adjusted for maternal age, pre-pregnancy BMI, maternal education, IVF, FPG, 2-h blood glucose. The results were presented with an adjusted odds ratio, aOR (95% CI);

IOM, Institute of Medicine; GWG, gestational weight gain; HDP, hypertensive disorders of pregnancy; PROM, premature rupture of the membranes; SGA, small for gestational age; LGA, large for gestational age; OR odds ratio; CI, Confidence interval

^a^*P* < 0.01

^b^*P* < 0.05,compared with the within IOM guideline subjects

### Association of rate of GWG in the second trimester of pregnancy with APOs among women with GDM

To further evaluate the effect of rate of GWG in the second trimester of pregnancy on APOs among women with GDM, 667 women were included for this analysis. Women with rate of GWG above the IOM guidelines were associated with a higher risk of HDP (aOR = 2.55; 95% CI 1.86–3.38), preeclampsia (aOR = 2.28; 95% CI 1.21–3.81), macrosomia (aOR = 1.20; 95% CI 1.02–1.82), and LGA (aOR = 1.42; 95% CI 1.24–1.97) than those with rate of GWG within IOM guidelines.

On the other hand, GDM patients with rate of GWG below the IOM guidelines were associated with a lower risk of macrosomia (aOR = 0.66; 95% CI 0.52–0.78) and LGA (aOR = 0.71; 95% CI 0.51–0.82). There were no statistically significant differences in the odds ratios of cesarean delivery, PROM, postpartum hemorrhage, fetal distress, preterm birth, and SGA among all three groups of women with GDM (Table 4).

**Table 4:** Association of adverse pregnancy outcome among gestational diabetes mellitus with IOM guideline on rate of GWG in second trimester of pregnancy

	Within (*n* = 243)	Below (*n* = 225)			Above (*n* = 199)		
	n(%) (Reference)	n (%)	crude OR (95% CI)	Adjusted OR (95% CI)	n (%)	crude OR (95% CI)	Adjusted OR (95% CI)
Caesarean delivery	99(40.7)	88(39.1)	0.85(0.72–1.21)	0.69(0.59–1.11)	86(43.2)	0.95(0.72–1.43)	0.83(0.69–1.32)
HDP	13(5.3)	10(4.4)	0.62(0.39–1.13)	0.60(0.36–1.16)	31(15.6)	3.25(1.74–3.88)^a^	2.55(1.86–3.38)^a^
preeclampsia	6(2.4)	4(1.8)	0.63(0.46–1.35)	0.74(0.41–1.45)	15(7.5)	2.51(1.59–3.67)^a^	2.28(1.21–3.81)^b^
PROM	103(42.4)	102(45.3)	1.08(0.81–1.48)	1.06(0.74–1.38)	81(40.7)	0.91(0.56–1.53)	0.82(0.47–1.41)
Postpartum hemorrhage	22(9.1)	24(10.7)	1.15(0.76–1.89)	1.09(0.69–1.71)	19(9.5)	1.08(0.45–1.77)	1.10(0.51–1.63)
Fetal distress	71(29.2)	61(27.1)	0.90(0.72–1.33)	0.86(0.74–1.19)	55(27.6)	0.92(0.71–1.20)	0.94(0.71–1.23)
Preterm birth	39(16.0)	43(19.1)	1.19(0.73–1.72)	1.11(0.64–1.65)	29(14.6)	0.893(0.45–1.49)	0.78(0.40–1.28)
Macrosomia	42(17.7)	15(6.6)	0.51(0.39–0.67)^b^	0.66(0.52–0.78)^b^	45(22.6)	1.40(1.27–2.10)^b^	1.20(1.02–1.82)^b^
SGA	18(7.4)	20(8.8)	1.23(0.84–1.77)	1.12(0.72–1.61)	10(5.0)	0.89(0.61–1.13)	0.90(0.79–1.01)
LGA	58(23.9)	17(14.2)	0.63(0.43–0.71)^b^	0.71(0.51–0.82) ^b^	71(35.7)	1.61(1.43–2.15)^b^	1.42(1.24–1.97)^b^

Multivariate analyses were adjusted for for maternal age, pre-pregnancy BMI, maternal education, IVF, FPG, 2-h blood glucose. The results were presented with an adjusted odds ratio, aOR (95% CI);

IOM, Institute of Medicine; GWG, gestational weight gain; HDP, hypertensive disorders of pregnancy; PROM, premature rupture of the membranes; SGA, small for gestational age; LGA, large for gestational age; OR odds ratio; CI, Confidence interval

^a^*P* < 0.01

^b^*P* < 0.05,compared with the within IOM guideline subjects

### Association of rate of GWG in the third trimester of pregnancy with APOs among women with GDM

We then analyzed the association between rate of GWG in the third trimester of pregnancy and APOs; 738 women with GDM were included for analysis. Women with GDM with rate of GWG above the IOM guidelines in the third trimester of pregnancy were associated with a significantly increased risk of cesarean delivery (aOR = 1.48; 95% CI 1.16–2.34), HDP (aOR = 1.93; 95% CI 1.08–2.98), preeclampsia (aOR = 2.17; 95% CI 1.35–4.37), macrosomia (aOR = 2.02; 95% CI 1.51–2.64), and LGA (aOR = 1.79; 95% CI 1.51–2.54). This group was also associated with a significantly decreased risk of PROM (aOR = 0.51; 95% CI 0.40–0.67) and preterm birth (aOR = 0.51; 95% CI 0.37–0.72).

GDM patients with rate of GWG below the IOM guidelines were associated with a significantly increased risk of preterm birth (aOR = 1.52; 95% CI 1.12–2.05) and SGA (aOR = 1.21; 95% CI 1.10–1.69). This group was also associated with a significantly decreased risk of macrosomia (aOR = 0.52; 95% CI 0.39–0.63) and LGA (aOR = 0.71; 95% CI 0.51–0.82) (Table 5).

**Table 5:** Association of adverse pregnancy outcome among gestational diabetes mellitus with IOM guideline on rate of GWG in third trimester of pregnancy

	Within (*n* = 258)	Below (*n* = 242)			Above (*n* = 238)		
	n(%) (Reference)	n (%)	crude OR (95% CI)	Adjusted OR (95% CI)	n (%)	crude OR (95% CI)	Adjusted OR (95% CI)
Caesarean delivery	90(37.0)	87(36.0)	0.85(0.74–1.23)	0.90(0.66–1.01)	122(51.3)	1.56(1.25–2.56)^a^	1.48(1.16–2.34)^b^
HDP	11(5.3)	9(4.0)	0.70(0.41–1.18)	0.63(0.41–1.11)	34(15.6)	2.27(1.15–3.32)^b^	1.93(1.08–2.98)^b^
preeclampsia	5(1.9)	5(2.1)	1.15(0.69–1.47)	1.07(0.60–1.32)	24(10.1)	2.30(1.47–4.53)^b^	2.17(1.35–4.37)^b^
PROM	127(49.2)	121(50.0)	1.08(0.87–1.39)	1.01(0.92–1.30)	73(30.8)	0.57(0.41–0.79)^b^	0.51(0.40–0.66)^b^
Postpartum hemorrhage	23(8.9)	22(9.0)	1.15(0.67–1.79)	1.08(0.62–1.71)	23(9.6)	1.35(0.75–1.77)	1.27(0.63–1.59)
Fetal distress	75(29.1)	68(28.1)	0.95(0.74–1.32)	0.94(0.70–1.25)	69(29.0)	0.99(0.83–1.21)	0.98(0.81–1.18)
Preterm birth	40(15.5)	78(32.2)	1.74(1.43–2.16)^a^	1.52(1.12–2.05)^b^	9(4.2)	0.42(0.33–0.64)^a^	0.51(0.37–0.72)^b^
Macrosomia	45(17.4)	14(5.7)	0.47(0.31–0.67)^a^	0.52(0.39–0.63)^a^	65(27.3)	2.19(1.65–2.83)^a^	2.02(1.51–2.64)^a^
SGA	18(6.9)	30(12.4)	1.32(1.23–1.84)^b^	1.21(1.10–1.69)^b^	9(3.7)	0.74(0.48–0.95)^b^	0.86(0.52–1.27)
LGA	62(24.0)	31(12.8)	0.56(0.42–0.71)	0.67(0.55–0.79)	100(42.0)	1.97(1.58–2.73)^a^	1.79(1.51–2.54)^a^

Multivariate analyses were adjusted for for maternal age, pre-pregnancy BMI, maternal education, IVF, FPG, 2-h blood glucose. The results were presented with an adjusted odds ratio, aOR (95% CI);

IOM, Institute of Medicine; GWG, gestational weight gain; HDP, hypertensive disorders of pregnancy; PROM, premature rupture of the membranes; SGA, small for gestational age; LGA, large for gestational age; OR odds ratio; CI, Confidence interval

^a^*P* < 0.01

^b^*P* < 0.05,compared with the within IOM guideline subjects

## Discussion

GDM is an abnormal glucose metabolism diagnosed and one of the common complications during pregnancy [24]. Some studies have reported the prevalence of GDM ranges from 18.1 ~ 41.4% based on IADPSG criteria [25], and the prevalence of GDM (6.1%) in this study was lower than previous studies, which may be related to the geographical differences, lifestyle changes, and lack part of 75 g OGTT. GDM and GWG have been previously reported to be associated with APOs [26, 27]. Considering the conflicting data regarding the relationship between inadequate GWG and APOs in women with GDM and the limited research on the association of the adequate range of GWG at different stages with APOs in GDM, we conducted a retrospective analysis among 1606 pregnant women with GDM. We showed the association between IOM guidelines for GWG, both in total and in the second and third trimesters of pregnancy, and APOs in women with GDM. In the present study, 33.9% of GDM women presented with a total GWG below the IOM guidelines, and 31.2% presented with a total GWG above the IOM guidelines. In a previous Chinese study, the rate of insufficient total GWG (29.6% and 12.5%) were found to be lower than our results [13, 20]. This variation may be due to rigorous lifestyle improvements, including nutritional therapy and exercise, leading to a leaner population in our study.

We then analyzed the associations of APOs with total GWG in women with GDM during pregnancy. Our results show that total GWG above the IOM guidelines increased the risk of LGA, macrosomia, cesarean delivery, HDP, and preeclampsia. Our findings were in agreement with several previous reports [26, 28–30]. Gou et al. showed that excessive GWG increased the OR for LGA and macrosomia [20]. Komem et al. demonstrated that total GWG above the IOM guidelines is related to cesarean delivery and LGA in women with GDM [26]. Furthermore, Cheng et al. performed the largest trial to date to retrospectively analyze data among women with GDM, which showed a remarkable risk for cesarean delivery, macrosomia, and LGA among women with GWG above the IOM guidelines [31]. However, Scifres et al. reported that women with both excessive GWG and insufficient GWG had a higher risk for macrosomia, which may be due to a different grouping method [32]. In addition, Cheng et al. showed that women with GWG above the IOM guidelines had a high risk of preterm birth [31]. Huang et al. found that, in general, pregnant women with both insufficient and excessive GWG had a higher risk for preterm birth [13]. In the present study, we showed that pregnant women with total GWG above the IOM guidelines had a lower risk of preterm birth, while pregnant women with total GWG below the IOM guidelines had an increased odds of preterm birth. These findings suggest that reasonable GWG among women with GDM may shorten the incidence of preterm birth. In concordance with other reports, our results also showed that women with total GWG below the IOM guidelines had a decreased odds of macrosomia, with an increased odds of preterm birth and SGA [16, 20, 21]. In contrast, Gou et al. showed that insufficient GWG did not increase the risk for SGA [20].

Recent studies reported the influence of GWG in the second and third trimesters of pregnancy on the incidence of APOs [33, 34]. For example, Bouvier et al. found that women with GWG above the IOM guidelines had an increased odds of HDP, cesarean delivery, macrosomia, LGA, and hypoglycemia in the second and third trimesters of pregnancy [33]. Wu et al. calculated GWG ranges using receiver operating characteristic (ROC) curve analysis (ROC targets) in a retrospective cohort study of women with GDM in Shanghai, China. They showed that ROC targets that provide better GWG guidelines during the second and third trimesters could improve pregnancy outcomes [35]. However, studies on the association of GWG in the second and third trimesters in women with GDM with APOs are limited. Thus, in the present study, we further analyzed the effect of IOM guidelines for rate of GWG in the second and third trimesters of pregnancy on APOs among women with GDM. Our results showed that rate of GWG above the IOM guidelines in the second and third trimesters of pregnant women with GDM was associated with a higher risk of HDP, preeclampsia, macrosomia, and LGA. LGA has been reported to be associated with excessive weight gain in the second trimester of women with GDM in a previous Brazilian cohort study by Drehmer et al., which confirms the results of our study [36]. Drehmer et al. also found that insufficient GWG in second trimester associated with SGA. In another retrospective observational study in India, Kashyap et al. found that pregnant women who had poor rate of GWG in second trimester were at an increased risk of SGA [37]. However, in our study, there were no statistically significant differences in the odds ratios of SGA in the below or above IOM guidelines group. In addition, we found that women with rate of GWG above the IOM guidelines in the third trimester of pregnancy were associated with a significantly decreased risk of preterm birth. However, our findings showed that women with total GWG below the IOM guidelines were associated with a significantly increased risk of preterm birth, which is in contrast with a previous report [36]. A fairly large body of literature on mechanisms linking GWG to preterm birth had been reported, a lower rate of GWG during pregnancy is associated with an increased risk of preterm delivery, and that a slow rate of gain during the latter part of pregnancy may be particularly important [38]. The inconsistency may be due to the different study populations and the adjusted confounding variables. Our findings on the relationship between APOs among women with GDM and IOM guidelines for GWG in the second and third trimesters may influence clinical practitioners to pay more attention to the control of GWG.

The study has several strengths. First, this study included a relatively large sample size and we adjusted for confounding factors to ensure reliable assessments. Second, we comprehensively analyzed the associations between IOM guidelines for GWG both in total and in the second and third trimesters of pregnancy with APOs in women with GDM, which has rarely been researched previously.

Our study however had several limitations. First, this study was limited due to the retrospective design. Second, some unmeasured confounders including smoking, diet, physical activity, and other factors were not assessed; therefore, the influence of these factors on APOs could not be explored. Third, since our study did not record weight when GDM was diagnosed, we did not investigate the influence of GWG on APOs specifically after the diagnosis of GDM.

In conclusion, our research suggests that GWG above and below the IOM guidelines, both in total and in the second and third trimesters of pregnancy, is a risk indicator for adverse obstetric outcomes in women with GDM. These findings suggest that it is essential to not only maintain an adequate total GWG during pregnancy, but also in the second and third trimesters among pregnant women with GDM. We hope to encourage physicians to deal with GWG using the IOM guidelines and to trigger intervention when it is required, which should help to reduce APOs. Prospective multicenter clinical investigations will be needed to elucidate the potential role of GWG in APOs among women with GDM.

## Data Availability

The data that support the findings of this study are available on request from the corresponding author. The data are not publicly available due to privacy or ethical restrictions.

## Abbreviations

IOM: Institute of Medicine
GWG: Gestational weight gain
BMI: Body Mass Index
IVF: In-vitro fertilization
SD: Standard deviation
FPG: Fasting blood glucose
OGTT: Oral glucose tolerance test
HDP: Hypertensive disorders of pregnancy
PROM: Premature rupture of the membranes
SGA: Small for gestational age
LGA: Large for gestational age
OR: Odds ratio
CI: Confidence interval
